# ‘The War Made Me a Better Person’: Syrian Refugees’ Meaning-Making Trajectories in the Aftermath of Collective Trauma

**DOI:** 10.3390/ijerph18168481

**Published:** 2021-08-11

**Authors:** Lisa Matos, Pedro A. Costa, Crystal L. Park, Monica J. Indart, Isabel Leal

**Affiliations:** 1 William James Center for Research, ISPA—Instituto Universitário, 1149-041 Lisbon, Portugal; pcosta@ispa.pt (P.A.C.); ileal@ispa.pt (I.L.); 2 Department of Psychological Sciences, University of Connecticut, Storrs, CT 06269, USA; crystal.park@uconn.edu; 3 Graduate School of Applied and Professional Psychology, Rutgers University, New Brunswick, NJ 08901, USA; monica.indart@gsapp.rutgers.edu

**Keywords:** collective violence, survivors of war and trauma, forced migration, posttraumatic growth, student refugees, world assumptions, core beliefs, cognitive processing, qualitative research

## Abstract

The centrality of the collective to Syrian identity and the ability of war to disrupt community ties have led to significant violations of Syrians’ pre-war assumptions about themselves, the world, and their place in the world. Guided by the integrated meaning-making model, this qualitative cross-sectional study assessed Syrian refugees’ meaning trajectories through their reappraisals of the war, attempts to repair community-informed shattered meanings, and those processes’ outputs (i.e., meanings-made) and outcomes (i.e., perceived psychological adjustment). We conducted semi-structured cognitive interviews with 39 Syrian war-exposed adults living in urban communities across Portugal, most of whom were beneficiaries of higher education programs for refugees. Interviews were analyzed through thematic analysis. Results suggest that the war severely disrupted Syrians’ sense of collective self, and that they repeatedly engaged in search for meaning, appraisals of the war, and reappraisals of shattered beliefs, life goals, and sense of purpose, both during wartime and in resettlement. In Portugal, despite persistent negative beliefs about the collective and ongoing and distressing searches for meaning, participants’ lived experiences concomitantly informed positive meaning reappraisals, including progressive restoration of worldviews, new opportunities for self-realization, and newly-found purpose, leading to perceived psychological benefits and growth. These findings suggest that meaning-making is both a trajectory and a dynamic process, informed by place and sociopolitical context. Clinical work to facilitate adaptive meaning-making and meaning-informed psychosocial interventions that help restore refugees’ shattered beliefs about safety, predictability, trust, and belonging, may be helpful directions to promote positive psychological adjustment and improve long-term integration prospects in refugees.

## 1. Introduction

War and other forms of collective violence can threaten individual and community assumptions of safety, predictability, and control, disintegrate the fabric of society, and lead to the mass displacement of civilians from their communities and countries in search of safety [1,2]. In post-migration settings, refugees attempt to create narratives that allow them to make sense of the past as they adjust to life and negotiate places of safety and growth, both within their own exiled communities and in their new host communities [3,4]. The process of making meaning of past trauma and reappraising worldviews shattered by community-impacting events, if successful, can ease pervasive distress, lead to positive psychological adjustment, and improve long-term integration prospects [5]. Understanding perceived group belonging inform meaning-making trajectories of collective trauma is therefore essential to promote arriving refugee communities’ recovery and healing.

Since 2011, the Syrian civil war has caused the forced displacement of more than half of its 22 million pre-war population [6]. What started as a peaceful uprising against the Syrian regime turned into a full-scale, protracted war that led to the killing or disappearance of more than 500,000 people, the internal displacement of 6.7 million civilians repeatedly pushed by shifting front lines [7], and to over 5.6 million people fleeing the country in search of safety. The vast majority of the latter have remained in Syria’s neighboring countries in the Middle East and North Africa, while an estimated one million people have sought durable solutions in Western countries of resettlement, arriving as asylum-seekers, resettled refugees, and beneficiaries of other immigration statuses [8]. Prior to making the decision and having the opportunity to flee, Syrian civilians have reported being repeatedly exposed to extreme traumatic events, including bombings, forced confinement, imprisonment, torture, which were often compounded with severe deprivation of basic needs and other potentially meaning-defying wartime stressors and losses that defied pre-war assumptions about the world and shattered community ties [9,10]. For those who reach resettlement (The word “resettlement” is used throughout the study to encompass post-migration settings, regardless of individuals’ legal status on arrival and host program. Likewise, “refugees” and “refugee populations” are used interchangeably to refer to forcibly displaced persons outside of the country of origin), their pre-flight experiences are additionally aggravated by journeys to safety and subsequent settlement fraught with uncertainty, additional potentially traumatic events (PTEs) and stressors, and adjustment difficulties [11]. It is thus unsurprising that Syrian refugees evidence high incidence of psychological distress as a normal reaction to repeated extreme circumstances, with recent studies documenting rates of post-traumatic stress disorder (PTSD) between 23% and 83%, often co-morbid with depression and anxiety symptomatology [12]. And yet, as with other refugee populations and despite the severity of trauma, Syrian refugees are also expected to perceive psychological benefits from their past experiences [13].

Psychological adjustment in the aftermath of trauma has been posited to require cognitive adaptation efforts that enable individuals to restore or exceed pre-trauma levels of psychological functioning [14]. According to Park’s theoretical meaning-making model [15], individuals possess core beliefs about themselves, the world, and their place in the world, and have desired goals and values that inform their lives’ purpose. Together, beliefs, goals, and subjective sense of purpose comprise one’s global meaning. These central cognitive structures give people a sense of predictability about their lives and provide them with a lens through which to interpret life experiences. When faced with an event that is perceived as discrepant from their global meaning, individuals experience distress that can trigger attempts to find comprehensibility and assign significance to the event, thereby reducing trauma-induced suffering. This search for meaning may lead to reappraising the traumatic event in order to fit one’s global meaning or to adjusting beliefs and goals shattered by trauma. When successful, meaning-making can lead to positive changes in psychological functioning, which has been operationalized through such constructs as perceived posttraumatic growth [16], increased self-efficacy, well-being, or quality of life [17].

Although both searching for and finding meaning in the aftermath of trauma are essentially individual, intrapsychic processes, meaning-making also occurs on a relational level through discussions with others [18]. In populations exposed to collective violence, the meanings survivors assign to the event tend to be construed within the community, as individuals seek social support as well as the perspectives of others to interpret the event and its impact on themselves and the community [19,20]. This need for co-authored meaning reconstruction is especially salient in non-Western communities of a collectivist nature and can be fostered through adaptive within-group coping strategies that can help restore connectedness, trust, and safety in shattered communities [4,21]. As the field of trauma recovery moves toward considering the ability to make sense of the trauma as a central aspect of survivors’ healing [22,23], understanding meaning-making as socially construed provides opportunities for individual and community healing [19].

There have been important advances in meaning-making research with refugee populations, where investigators have tested some of its theoretical propositions, such as meanings-made of trauma, reappraisals of core beliefs, cognitive and emotional processing, or generational appraisals of collective trauma [24,25,26]. However, given the dynamic nature of the refugee experience, refugees’ recurrent susceptibility to meaning-defying PTEs, and propositions in the refugee trauma literature suggesting that meaning-making is an ongoing process with no defined end [20,27], it is important to investigate changes in meaning over time and consider the impact of place and circumstances of settlement on cognitive reappraisals of past experiences.

In the aftermath of the 2015 refugee crisis, Portugal committed to hosting unprecedented—despite symbolic in the larger European Union context—numbers of asylum-seekers relocated from Greece and Italy following Aegean and Mediterranean Sea crossings, as well as UNHCR resettled refugees [28]. By the end of 2019, a total of 2144 refugees arrived through these two mechanisms, with Syrians comprising one of the largest arriving groups. Following protracted exposure to armed conflict, complicated migration journeys, and settlement in communities across Portugal, it is important to understand if Syrian war-exposed individuals, despite being recently arrived, perceive positive psychological changes in resettlement and, if so, the cognitive processes that contribute to those changes, as well as how the resettlement experience contributes to perceived benefits.

Considering the centrality of the collective to Syrian identity, and the ability of war to disrupt community ties, this study aims to assess community-informed posttraumatic cognitive reappraisals, understood as socially construed meanings, in a sample of war-exposed Syrian civilians living in Portugal. To do so, we conducted an exploratory qualitative study to capture a diversity of meaning trajectories that focused on examining reappraisals of the Syrian war in resettlement, attempts to repair shattered meanings, and those processes’ outputs (i.e., meanings-made) and outcomes (i.e., perceived psychological adjustment).

## 2. Materials and Methods

### 2.1. Study Design

This study utilizes data from a larger research project that examines how survivors of the refugee experience make meaning of past experiences and achieve psychological adjustment in post-displacement settings. Guided by Park’s integrated meaning-making model [15], this cross-sectional qualitative study examined posttraumatic cognitive reappraisals of PTEs and global meaning systems in Syrian war-exposed adults through semi-structured cognitive interviews. Cognitive interviews corresponded to Phase 2 of the larger project, and the Arabic language research protocol had previously been tested during Phase 1 Focus Groups.

### 2.2. Participants

Participants were recently arrived Syrian adults living in urban communities across Portugal. Eligibility criteria included (1) having Syrian nationality or background (e.g., stateless Palestinian refugees from Syria), (2) being 18-year-old or older, (3) being in Portugal for a minimum of six months, and (4) being Arabic speaker with English or Portuguese fluency. Prospective participants were excluded if they manifested acute psychological distress or had apparent cognitive deficits. The sample was recruited across continental Portugal through a combination of intentional and snowball sampling. A total of 40 individuals were interviewed between January and May 2019, with one Kurdish man dropping out after becoming distressed during the interview. The final sample thus consisted of 39 participants: 20 men (51.3%) and 19 women (48.7%) between the ages of 19 and 37 (*M* = 27.1; *SD* = 4.8). Thirty-one individuals were student refugees (i.e., beneficiaries of higher education programs for refugees), five were relocated from Greece, and three were spontaneous asylum-seekers. Participants’ median length of stay in Portugal was 32.7 months (*SD* = 27.9), ranging from 11 to 67 months. Twenty-four student refugees travelled directly to Portugal largely from Beirut, whereas the remaining 15 individuals spent between two and 59 months in transit, with a mean of 23.2 months (*SD* = 18.3). Thirty-four participants were single (87.2%) and five (12.8%) were married. Eighty-four percent (*n =* 33) had some form of higher education, including doctoral (*n =* 2, 5.1%), master’s (*n =* 11, 28.2%) and bachelor’s (*n =* 18, 46.1%) degrees, and the remaining six (15.4%) had 12th grade education. Family-inherited religious background was: Muslim-Sunni (*n =* 16, 41.0%), Muslim-other/non-specific (*n =* 8, 20.5%), Alawite (*n =* 7, 17.9%), Ismaili (*n =* 2, 5.1%), Druze (*n =* 2, 5.1%), Christian-Catholic (*n =* 2, 5.1%), and Christian-Orthodox (*n =* 2, 5.1%).

### 2.3. Materials

The study protocol included: (1) a sociodemographic questionnaire developed for the purposes of the study and administered as a structured interview; and (2) a semi-structured interview designed to guide participants through posttraumatic reappraisals of beliefs, goals, and sense of purpose. The Syrian war was set as baseline for a pre- and post-trauma global meaning structure, and individuals were invited to reflect on changes to their cognitions and self through the opening question, “when you think back on your life before the war and now, can you describe how it changed?”. Subsequent questions invoked changes to specific aspects of global meaning. For example, on eliciting narratives about sense of purpose, the question “what was life about before the war?” invoked pre-trauma cognitions, with the following probes if participants appeared stuck: “what is it that you lived for before the war, and what is it that you live for now?”.

### 2.4. Procedures

Due to the target population being recently arrived, expected high exposure to extreme trauma and significant stressors, and reported research fatigue among the community, outreach strategies were iteratively discussed with, and mediated by, key community leaders and resettlement agencies. Arabic language information materials were disseminated through leaders’ and agencies’ informal networks, and through postings on the research project’s social media accounts (e.g., Facebook). Study materials were developed in English, forward translated to Arabic by a Syrian research consultant, and pre-tested in Focus Groups prior to data collection. To accommodate concerns expressed by participants during protocol pre-testing related to anonymity within the community, individual interviews were conducted without assistance of an Arabic language interpreter, in either English or Portuguese according to participant fluency. Print Arabic versions of all materials were available to participants. Detailed community-, trauma-, and ethics-informed findings from research protocol testing are published elsewhere [29].

All interviews were conducted by the Lisbon-based Lead Researcher (first author) who is experienced in interviewing survivors of refugee trauma, and who travelled to meet participants in the northern cities of Braga, Guimarães and Oporto (*n* = 18), in central Aveiro and Coimbra (*n* = 10) and the capital Lisbon (*n* = 10), and southern Évora (*n* = 1). Researcher and participants met in quiet places of participants’ convenience, namely university offices or city parks, for an average of 90 min, and interviews were audio recorded. Prior to enrolling, individuals were informed of the purpose of the study and the voluntary nature of their participation. Bounds of anonymity and confidentiality of information were reviewed, and individuals were given an opportunity to ask questions and clarify any doubts prior to signing consent forms. Although the purpose of the interview was not to narrate PTEs, there was potential for distress and retraumatization as participants reflected on past events and the impact of those experiences on their global meaning. The sample was thus instructed on the nature of symptoms and normal reactions to the retelling of their stories and informed of the possibility of being referred for pro-bono psychosocial support, as needed. At the end of the interview, participants were offered a €10 gift card. Interview follow-up included a message to check on participant wellbeing after revisiting their past for the purposes of the research. The study received ethics approval by ISPA—Instituto Universitário’s Ethics Committee (Ref. D/004/09/2018).

### 2.5. Data Analysis

Audio recordings were transcribed, participants’ names were replaced and coded, and transcriptions were analyzed using thematic analysis [30]. Park’s meaning-making model [15] provided the structure to analyze participants’ cognitive reappraisal trajectories in relation to the war as the collective traumatic event. To encompass an array of different perspectives, the data analysis team included refugee trauma and psychological adjustment researchers (first and fifth authors, respectively), one qualitative methodologies instructor (second author), and two research assistants (RAs) trained in thematic analysis. The first author conducted a first in-depth review of all interview transcripts and created the initial coding, which was preliminarily organized into potential themes that were reviewed and discussed with the co-authors. Using the preliminary thematic map, the RAs analyzed the data set, meeting regularly with the first and second authors to discuss discrepancies, reach consensus, and ensure consistency. Considering the scope of the study, themes were reorganized following the main components of the meaning-making model as a trajectory. Transcripts were examined to identify trajectories of community-informed meaning, i.e., cognitive processing narrated as a collective experience, in relation to the Syrian war as the overall stressor. Data were managed and analyzed using MAXQDA 20 software (Berlin, Germany: VERBI Software). In the context of the current study, themes pertaining to psychological distress or to individual reappraisal processes were excluded from the analysis. Throughout both phases of analysis, team members met regularly to review findings, refine themes and subthemes, and discuss narratives that deviated from the thematic map, which gave credence to the diversity of individuals’ meaning-making experiences.

## 3. Results

**Figure 1:**
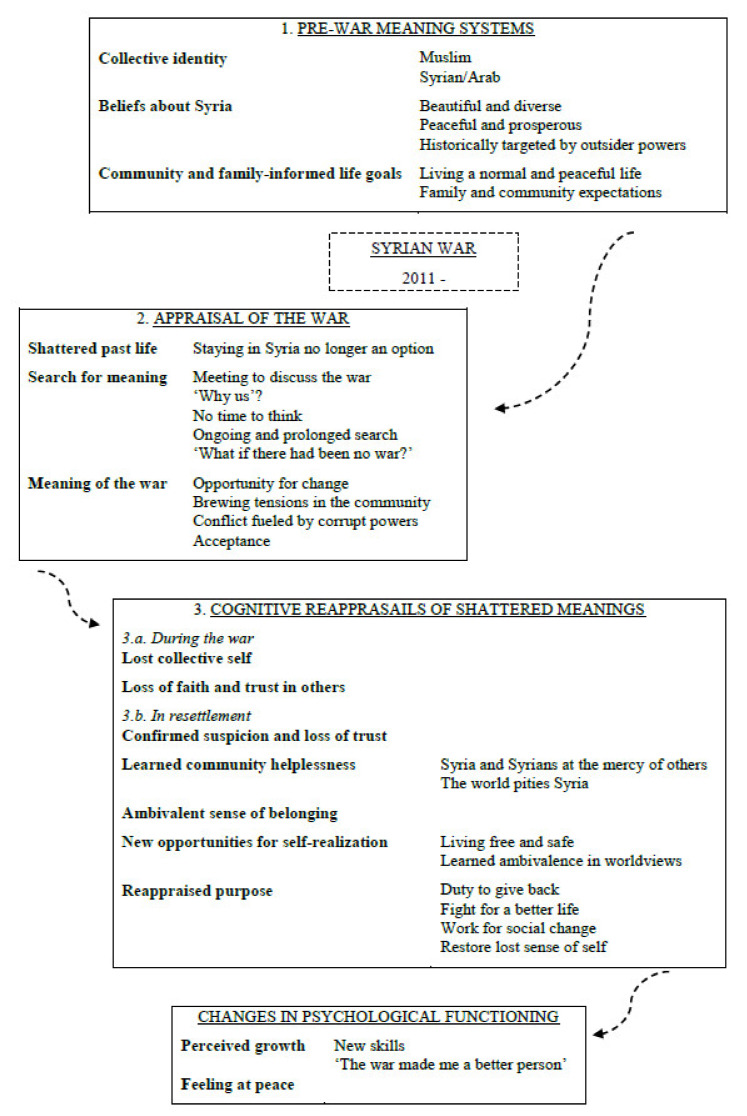
Thematic map.

Data were organized in four sections: pre-war meaning systems; appraisals of the war; cognitive reappraisals of shattered meanings; and changes in psychological functioning. Themes are discussed in each section and, where subthemes were identified, in the interest of text fluency, they are named in the introductory paragraph and subsequently examined as elements of the overall theme. Figure 1 shows the Thematic Map of identified themes and subthemes with arrows illustrating possible trajectories of cognitive processing.

### 3.1. Pre-War Meaning Systems

Participants spoke of their lives prior to the onset of the war and reflected on three themes of the collective self: Collective identity (subthemes: Muslim; Syrian/Arab), Beliefs about Syria (subthemes: Beautiful and diverse; Peaceful and prosperous; Historically targeted by outside powers), and Community- and family-informed life goals (subthemes: Living a normal and peaceful life; Family and community expectations).

#### 3.1.1. Collective Identity

Participants emphasized two intertwined aspects of their collective identity pertaining to religion and to their Syrian cultural identities. With a large majority Muslim sample, being “Muslim by heritage” (AK, woman, 31) was a sentiment expressed by several participants, who spoke of being raised in religious families that often conformed to cultural expectations. Despite her family not being “really religious”, 23-year-old GH reported that “we pray and everything like all Syrians”. Religion shaped identities and community relations: “on Friday, everyone goes [to the mosque] to meet their friends and talk with each other. They’re not going because of the religion” (BS, man, 27).

#### 3.1.2. Beliefs about Syria

The cultural context provided the structure through which to live and interpret life. Prior to the onset of the war, Syria was predominantly—proudly and longingly—recollected as beautiful and diverse, as well as peaceful and prosperous. As with many others, BM (woman, 26) spoke of wealth and prosperity, “everything was available to us, anything you can dream of, you could get before the war”, whereas KK (woman, 30) emphasized its cosmopolitan nature, “in Syria, the diversity is amazing! In Damascus, especially. You are walking down the street, you can see girls with skirts, girls with hijab, (…) the diversity is really amazing”. On the other hand, there were some participants who reflected on the cycles of violence in the region and Syria’s predestination to be targeted by other powers. One woman explained, “if you look at the history, you’ll see so much aggression. Wars and stuff. This is something that is transmitted over generations” (AK, woman, 31), and the sentiment was echoed in the words of another participant who stated, “we are under attack, always” (NM, man, 27).

#### 3.1.3. Community- and Family-Informed Life Goals

Participants admitted to rarely giving much thought to how they expected their lives to develop. Most envisaged living a normal and peaceful life, which included studying, getting a house, marrying, having children, etc., which one participant equated to living regular lives “like in Portugal right now” (AI, man, 25). He added: “we had our routines, go to work, university, meet our family and friends”. Life essentially conformed to family and cultural expectations, which MS (man, 32) equated to “[living] by inertia, moving with the culture, doing what everybody else did”.

### 3.2. Appraisal of the Syrian War

We examined participants’ accounts for perceived extent of threat, causes of the war, implications for the future of the community, and acceptance and integration of the event. Three themes were identified and are discussed below: Shattered past life (subtheme: Staying in Syria no longer an option), Search for meaning (subthemes: Meeting to discuss the war; ‘Why us [Syrians]?’; No time to think; Ongoing and prolonged search; and ‘What if there had been no war?’), and Meaning of the war (subthemes: Opportunity for change; Brewing tensions in the community; Conflict fueled by corrupt powers; and Acceptance).

#### 3.2.1. Shattered Past Life

“When the war started, everything changed” (BS, woman, 32). The onset of the war in 2011 caused severe violation of participants’ expectations of safety and predictability, and shattered life’s continuity. Participants drew a clear before-and-after the war line where pre-war lives were largely recalled with longing, and the growing violence and terror around them, forced confinements, and severe disruptions of their daily lives were retold with shock and disbelief. NM (man, 27) emphasized his alarm by stating: “I was living the maximum happiness I could. Going through something that threatened my life or losing things I used to [take for granted] was a big shock for me”. Participants discussed the sudden—for some—or progressive—for others—realization that what they had idealized for the future had ceased being an option, and that their ability to “complete” their lives in Syria was no longer a possibility. Flight decisions were informed by perceived imminent danger, men’s fear of being forcibly recruited into the army, collapsing systems and institutions and ensuing lack of future prospects, and realization of the community’s mass exodus. One participant explained that, although she had always considered the possibility of one day studying in Europe, with the war, that was “no longer just an option, it’s a need. What small opportunity you had to stay [in Syria], is now gone. You need to go” (ME, woman, 30).

#### 3.2.2. Search for Meaning

The extreme nature of the conflict and repeated challenges to pre-war assumptions triggered in Syrians the need to make sense of the events around them. Because the experience was not just personal, it needed to be examined and interpreted by and within the collective. In our sample, participants reported meeting in small groups to discuss the war, whether within the family or with their friends and others in the community, and, as such, attempting to understand what was going on and find a narrative that helped fit the war into their worldviews. BS (woman, 32) explained: “we had deep discussions about these things. In our small places, we could analyze what was going on. We noticed that it was a big countries game”.

A common formulation as individuals sought comprehensibility amidst the war entailed asking why, articulated not just in the singular (i.e., why me?), but in the plural (i.e., why us?). “What was happening to us raised a lot of questions”, painfully recalled AG (man, 36), “why? Why us? Why me? Why my family? Why are these things happening?”. Other ruminative formulations included questioning the fairness and justice of it all, which, in a majority-Muslim country, included reaching out to God and examining their religious beliefs. KK (woman, 30) observed: “with the war, people started to lose their faith. Everybody started asking, ‘why is this happening? Oh God. Where’s God?’ Just like, ‘if there is a God, He would not allow this to happen’”.

As the war expanded and became protracted, these ruminative processes appeared to subside or be purposefully postponed as individuals and the community focused on survival and the daily challenges of life in wartime. One man described the barrage of information and exposure to extreme events on a daily basis that led him to question his pre-war beliefs about the community:

“At the time, I didn’t have time to ask myself [why], because we were just receiving information, watching the news, trying to understand what was going on, why people are getting killed, disappearing, why people became zombies suddenly and started killing others. This was hard for me because we were always a country where people loved each other.”

(NM, man, 27)

The narrative that had sustained individuals during the challenges of the war was subsequently challenged by the compounding forced migration experience, which appeared to reactivate searches for comprehensibility and significance. There were some participants who shared distressing accounts of wanting to find purpose in their experiences, often amidst significant living difficulties in Portugal. Filled with sorrow, RA (woman, 36) stated: “We were forced to leave our country. The future we were building there. [Others] didn’t have to leave their countries. They didn’t go through these things”.

In trying to find worth in their suffering, one final element in the search for meaning emerged, as some participants shared occasionally contemplating what their lives would have been as if had there been no war. Our sample was largely composed of individuals who were adolescents and coming into adulthood as the war broke, which led participants to reflect on their developmental time and how the conflict had shaped their sense of self. ME (woman, 30) explained: “let’s say in a virtual reality there was no war. Would I have developed the same way or am I this way because of war?”. The forced migration experience compounded by forced separation from loved ones led YK (woman, 25) to wonder what life would have been like if had had she been born in Portugal, a country with no war, where she could be “with [her] family, and all [her] friends”. She added: “what if I didn’t have to separate from anyone I love?”.

#### 3.2.3. Meaning of the War

The war began with an uprising within the larger regional context of the Arab Spring and was largely welcomed as an opportunity for change. There was a shared community purpose and hope that the uprise would lead to needed “change [in] the institutions, change [in] the mentality,” (SH, man, 37). In a hope- and excitement-filled account, AK (man, 24) explained how the revolution made room for needed growth and knowledge in the society: “there were really good ideas. People teaching each other about civil society, separation of religion and state laws, how we can have human rights. All these human rights that we had no idea about!”.

However, as the violence escalated, the need to create narratives that helped Syrians make sense of the war increased. Many participants initially appraised the war as being the result of brewing tensions in the community and fueled by corrupt powers. Despite living in peaceful coexistence, as some knew, and others realized, “there was a sort of aggression between Syria’s sub-communities that was there for years, which no one had addressed” (SH, man, 37). As the conflict grew and worsened, participants spoke of realizing that they were pawns in a game controlled by political and religious powers that fomented discord in the community. Even if unable or unwilling to assign blame beyond naming an overpowering “they”, YK (woman, 25) expressed her frustration as follows: “they made people fight against each other. (…) And it’s a game. I don’t know the rules of this game, but it’s a dirty game. And they are playing with souls”. The process of uncovering the perceived truth evidenced by war was lengthy for some, with NM (man, 27) admitting that it had “[taken him] a long time to realize the bad things the regime was doing”, whereas for a number of others watching “the army shooting people” (MA, man, 30) was the trigger for their own realization. Especially concerning in a majority-Muslim society was the perception, by the community, of the part religious groups and leaders, both in Syria and elsewhere in the Arab world, played in the war. AK (woman, 30), who was then a practicing Sunni Muslim, stated: “we started to see—me and the community, my friends, my family—how religious leaders were involved in the conflict. So we lost trust in these people”.

Despite perceiving significant discrepancies between the events around them and their pre-war beliefs about Syria and their community, some participants were able to integrate and narrate the war as inevitable, and accommodate it within cyclical, generational violence. Several individuals provided similar narratives of regional geopolitics, where their homeland was perceived as a target. SS (woman, 28) stated: “we were raised to know that we were living near the enemy, which is Israel. So, we always knew that there was going to be a war. Now, later, it’s going to happen”. Despite the preparedness for war, the escalating violence defied expectations. MK (man, 22), who at the beginning of the war was 14 years old, promptly referred back to family-inherited Syrian history: “I understood [what was happening] right away, because my parents went through something similar in the 1980s. Similar, but not as devastating”.

Lastly, as the war progressed, it overwhelmingly became too absurd to comprehend, and yet something with which they had to learn to live. In their youth and despite living under constant threat, numerous individuals recounted defying parental instructions to stay home, accepting the possibility of death, as well as painfully realizing that they had learned to accept and live with the horrors of war: “The first time I saw people dead was hard. But the worst was when I *got used to* [emphasis added] seeing people dead” (NM, man, 27).

### 3.3. Cognitive Reappraisals of Shattered Meanings

“I think the war plays a big role in questioning what you believe in”. With this statement, MA (man, 27) insightfully captured the meaning-making processes triggered by the war as an event that overwhelmed individuals’ cognitive structures. Wartime trauma and daily stressors, and the difficulties associated with displacement were repeatedly appraised against participants’ global meaning structures. We identified two themes of wartime meaning reappraisals—Lost collective self and Loss of faith and trust in others—and five themes of reappraisals in resettlement: Learned suspicion and loss of trust; Learned community helplessness (subthemes: Syria and Syrians at the mercy of others; and “the world pities Syria”); Ambivalent sense of belonging; New opportunities for self-realization (subtheme: Learned ambivalence in worldviews); and Reappraised purpose (subthemes: Duty to give back; Fight for a better life; Work for social change; and Restore sense of self).

During the War

#### 3.3.1. Lost Collective Self

The war was understood as having created or evidenced tensions along religious and political lines, which were hard to reconcile with pre-war community beliefs and sense of a cohesive collective. As neighbors and colleagues turned on each other, “you realize that something was wrong from the beginning” (MZ, man, 28), and that Syrians’ previously harmonious lives were, in fact, “a lie”. As with AK (man, 24), many felt that the collective self had been artificially held together through repression: “we lived in a lie. That we are a unity, we are one piece. Actually, people hated each other, inside, deeply. We were just not allowed to say it”. Whereas others longed for their lost beloved community:

“We started to feel, ‘oh, he is Sunni, he hates me, and I hate him.’ It’s a very bad thing. Because we have never been like this in Syria. We live with Christians and Muslim and Kurdish and every religion, and we were very happy. And I think every person agrees with me. In Syria, we were very happy, but I don’t know what happened…”

(BM, woman, 26)

#### 3.3.2. Loss of Faith and Trust in Others

As the fabric of the community disintegrated, generalized suspicion took hold. Participants shared deep, sorrow-filled accounts of losing faith in their leaders and losing trust in each other. “There was a feeling of having lost the trust we had”, as GH (woman, 23) put it. She added, “after the [onset of the] war, I lost faith in my friends”, which carried over into Syrians’ lives in exile: “I believe that people now have a problem trusting each other”.

In Resettlement

In resettlement, years after initial post-war reappraisals, participants spoke at length about changed beliefs, goals, and purpose, informed by their new setting.

#### 3.3.3. Suspicion and Loss of Trust

In Portugal, the war continued to be understood as the result of the will of the regime and religious groups, as well as outside powers. With some time and geographic distance, some participants assigned blame squarely on their own community and culture. “We created the war!” decried AK (woman, 31), “we have to confess that we are responsible for this damage. We throw responsibility around, but this is craziness! We are an integral part of this war. Our mentality, our vision, our way…” Suspicion and distrust therefore continued to severely disrupt community relations. Despite well-intended efforts by host organizations to create spaces for community dialogue and healing, with unresolved tensions and the war still active, Syrians did not appear equipped to engage in fruitful dialogue: “we had summer school this year in my university and there were a lot of Syrians—the theme was “rebuilding Syria”—and all the time people were arguing and fighting. There was all this tension because of the war” (GH, woman, 23).

#### 3.3.4. Learned Helplessness

Perceived community helplessness derived from the realization that the still ongoing war was fueled by geopolitics, where outside countries continued to dictate the fate of Syria—“[the war] is not in the hands of Syrians anymore. Maybe we started it, but we can’t finish it now” (MA, man, 24)—, and of Syrians—“my dream was to study in a US university, because they are the best in the world. But then I lost my scholarship. I believe they didn’t issue me a visa because I’m Syrian” (MW, woman, 32). As nationals of the largest refugee-source country, Syrians are often depicted for their vulnerabilities and weaknesses, which was largely shown to humiliate and hurt participants’ sense of pride. “Before the war, nobody heard about Syria, but now everybody talks about us, poor people”, explained BS (woman, 32). SS (woman, 28), who gave unwavering accounts of the strength of her people and pride in her community and heritage, related feeling hurt when she heard other Syrians in exile say that they do not want to go back after the war:

“Okay, I know our country is not that good and maybe you don’t want to go back, but don’t say it. Because when other people hear it, they will say, ‘what kind of country is it that their own people want to leave it?’”

#### 3.3.5. Ambivalent Sense of Belonging

As participants attempted to reconcile past lives and shattered identities with the opportunities before them in the new country, they struggled with understanding where they now fit in. This theme was characterized by ambivalence and a diversity of experiences, where most participants were still reconciling their loss with their newly found affiliations, often negotiated within family and community expectations. BS (man, 27), who had grown used to the European and Portuguese cultures and struggled to imagine the possibility of adjusting back to life in Syria, shared a recent conversation with his mother. As they revisited the delicate subject of his potential return, she replied: “‘who said you will ever come back?’ She was prepared for it! She wants me to come back, but she [finally understands] that it’s better to live here”. Others, such as student refugee ME (woman, 30), conveyed distressing accounts of lost belonging. After traveling back to Syria for summer break, ME recounted: “I came here [to Portugal] and didn’t fit in. So I always thought that when I go back [to Syria] I would fit in more. But I went there and I didn’t. Now I don’t fit anywhere, and I don’t know what to do”. Lastly, an important few reported opposite-end allegiances, be it finding, in Portugal, their new home, be it through strengthened allegiance to the homeland. SS (woman, 28) declared: “I feel more connected to my homeland! (…) My [Portuguese] boyfriend knows that, for me, Syria is my final destination”.

#### 3.3.6. New Opportunities for Self-Realization

Students in our sample shared guilt-laden accounts of attempting to resolve the discrepancy between the destruction caused by the war and the opportunity the war provided for them to study abroad. They struggled with the benefit they derived from all the destruction: “there are opportunities because of the war. I will not say that’s a good thing, but it’s one thing that would never have happened without the war” (AO, man, 23). As participants discussed negotiated identities and place in the new country, they narrated rich accounts of the possibilities offered by the resettlement setting, with many talking about the ability to live free and safe in Portugal. AO (man, 31) reported telling his fiancée back home that “we can have a good life [in Portugal], find a good job, and feel safer”. Coming from a majority Sunni-Muslim country with strict rules about women’s behavior, some young women passionately shared experiences of newly found freedom and identity. KK (woman, 30) stated:

“I survived a controlled society. I feel like I had one life before and now I have a new life. So, I survived a society where they judge people and judge girls for what they wear. And now I am free.”

When reflecting on beliefs of justice, kindness, or perceived control over one’s life, participants seldom gave unequivocal answers. As one woman put it when asked if the world was fair, “it’s like you’re asking me if the world is black or white. I can’t answer that question. It’s a really wide range of colors!” (AK, woman, 31). This type of thought process reflected not only the complexity of participants’ experiences, but also evidenced layered meaning systems. There was ambivalence in worldviews regarding safety (e.g., “[the world is safe] depending on the country you live in, the people you live with, and the conditions of your life.”; BM, woman, 26), benevolence (e.g., “there are bad people who make the world worst, but there are always good people who help others go through difficult times.”; HH, man, 36), and fairness (e.g., “you don’t think about what is fair or not fair. You have to think: ‘I have this [scholarship] opportunity and I have to take care of myself. Nobody else will do it.’”; MS, man, 32).

#### 3.3.7. Reappraised Purpose

Life in resettlement provided a unique opportunity to rebuild their lives in peace and safety, as well as to restore individuals’ sense of purpose. Because the majority of participants were working towards graduate and undergraduate degrees, in our sample, subjective purpose accounts were often informed by their student refugee identities. “I’m here studying because war happened in my country, because people suffered, and this is a responsibility that I don’t take lightly. I cannot remove this from who I am, and I have to give something back” (AO, man, 23). This feeling was shared by many students, who struggled to reconcile the guilt they felt for their privilege as beneficiaries of student programs, an overwhelming sense of responsibility to succeed, and a perceived obligation to work towards Syria’s post-war reconstruction and healing. In the privacy and anonymity of the research interview, however, a few students guiltily admitted to having changed their original plans of return: “I don’t think I’m going back. Honestly, I’ve been thinking about bringing my sisters. Here we can build a better future. I’m really sad and I’m sorry for that, but we have to think about our future” (BM, woman, 26).

Having lived through the unpredictability of war and displacement, many participants learned to be cautious about the future and to focus on immediate, tangible goals. As some envisioned a future in Portugal, their lives were now guided by the need to prove themselves to the host community: “the Portuguese gave me this opportunity of a lifetime, and I need to prove to them and to the world that I deserve it” (HH, man, 36). In the case of others, after all the struggles and suffering, their current purpose was informed by survival and restoration of their pre-war self: “I want to find a job and be totally independent. Focus on myself, my efforts, my skills. This is my new goal: to return again to who I once was” (AK, woman, 31).

### 3.4. Changes in Psychological Functioning

Although the lessons learned from the war and the refugee experience led participants to report often feeling anxious and depressed, with a small number of them reporting clinically-significant posttraumatic stress disorder (As part of the larger research study, participants in this sample completed the Harvard Trauma Questionnaire—Arabic Version [31]. Findings pertaining to prevalence of trauma and PTSD are reported elsewhere [9]), participants also retold accounts of improved psychological functioning. There were two themes of positive psychological adjustment: Perceived growth (subthemes: New skills; and ‘The war made me a better person’; and Feeling at peace).

#### 3.4.1. Perceived Growth

In our largely young, single, student sample, the war and the subsequent safe and purpose-informed settlement in Portugal created opportunities for self-affirmation and growth. There were abundant accounts of increased courage, strength, and kindness. RN, a recently arrived 19-year-old woman explained, “I think that these events were good for me, because without the war I couldn’t be a strong woman”, whereas another attested to having “more appreciation for life now” (YK, woman, 25). Another spoke of the newly acquired freedom and “courage to talk about [her] beliefs” (KK, woman, 31). As beneficiaries of higher education programs for refugees, several students and former students also perceived benefits from the new skills acquired. AO (man, 31) summed it up and spoke of optimism: “here I learned languages, I have new skills, and now I’m working. So, I look at the future in a really optimistic way”.

The second subtheme was characterized by especially moving and love-filled narratives: “you feel that you need to be with people who really love you, who really support you. (…) The war made us more emotional towards each other, more supportive of each other”, recounted YK (woman, 25). As she integrated the ambivalence of reappraised worldviews and spoke of learned unfairness, RJ (woman, 27) added: “at the same time [the war] makes you a better person, because you suffered a lot and you do not want someone else to suffer. So, yes, I feel motivated to do more and be better”.

#### 3.4.2. Feeling at Peace

As they wrapped up their interviews, a few participants shared pride-filled accounts as to how far they had come in the aftermath of a war and forced displacement. They narrated ways in which they had been able to integrate their past into their rebuilt selves. When facing bouts of sadness or distress, AK (man, 24) reminded himself to think “rationally”. He explained, “‘rationally’ means respecting the experience I had. (…) It built me. I’m a different person. I told you, I’m proud of this journey”. Despite their youth, they also gave insightful narratives of healing as a journey fostered by safety, peace, and kindness in the host country:

“When I feel depressed, I close my eyes and remind myself that I’m in Europe and that I am actually working on my dreams. So, I always remember that. (…) People here [in Portugal] surround us and they are very good with us so we can feel more at peace. So right now, I feel like I’m healing.”

(DA, woman, 24)

## 4. Discussion

Understanding meaning-making processes in the aftermath of collective violence as relational and informed by culture, community, and place is essential to advance evidence-based practices of trauma recovery and healing in refugee populations [7,18,32]. This study examined meaning appraisals as collectively and socially construed, in a sample of Syrian refugees living in Portugal. Although we used a pre-war/post-migration structure in the interviews to access changed cognitions, most participants were able to provide rich accounts of their journeys in meaning: from pre-war assumptions about the community and the collective self, through initial and evolving appraisals of the war while in Syria, perceived violations of and discrepancies from pre-war community assumptions, revisited need to seek comprehensibility and significance, and wartime reappraisal of shattered meanings. Once in Portugal, meaning-making narratives were revisited with the distance allowed by time, and informed by the new settlement and community, new stressors, and individuals’ lived experiences. These narratives contributed to concurrent positive and negative meanings-made and subsequently to posttraumatic psychological adjustment.

Our sample was generally young, with participants coming into late adolescence/early adulthood at the onset of the war, and also highly educated. The latter was unsurprising considering Syria’s massive investment in higher education opportunities, especially for women and in the fields of science and technology [33]. Albeit not an intentional recruitment strategy, the majority of participants were graduate and undergraduate students arriving under the Portuguese Government’s Global Platform for Syrian Students (GP4SYS), which was created in 2013 to provide safe access to higher education opportunities for Syrians affected by war [34]. The inclusion of war-exposed individuals regardless of refugee and derivative statuses enriches our understanding of the diversity of arriving communities’ lived experiences and counters all-encompassing and constraining legal categories that may silence individual and sub-group perspectives [4]. This is especially important for Syrian forced migrants, whose multiple group identities and belongings have been shown to be protective and to help provide a sense of identity continuity in exile [35].

Participants’ meaning-making trajectories were examined in relation to the war as the overall collective stressor, despite individual exposure to different types of potentially shattering trauma and losses and focused on perceptions of the collective self and community functioning. Pre-war beliefs were often prefaced with “we always knew that”, followed by aligned statements about regional geopolitics, which offered an initial narrative structure into which to integrate the war. However, as the conflict expanded and violence intensified, previous assumptions about a unified collective were violated and perceived as flawed and maintained by a repressive regime that quieted dissent [36]. In our study, the overall forced migration aspect of participants’ experience was painfully articulated as losing an imagined future in Syria, which required reworking shattered life goals that were informed by community and family expectations, and thus simply described as living “normal lives”. As proposed by the meaning-making model, the war was progressively perceived as discrepant from pre-2011 beliefs about the country and the collective self, prompting, in Syrians, the need to make sense of the events. Search for meaning entailed two processes—search for comprehensibility and search for significance—which have been posited to occur sequentially [15], but may, as we observed, not only be concurrent but also reinitiated and revisited with displacement and new social context. Participants’ initial appraisals of the war often entailed relational processing through, likely adaptive [7], small-group discussions with family and friends where they attempted to build a narrative that helped explain and integrate the conflict. This need for comprehensibility was often triggered through plural “why us?” formulations, as individuals sought to understand the implications of the war, not just for themselves but for the community. This finding aligns with previous studies with refugee populations reporting analogous community-informed verbalizations [20].

One salient aspect of this search pertained to the urge many felt to see worth in the war and in their post-war lived experiences. This intentional cognitive reframing evidenced the disruption the war had imposed on prior expectations of continuity [25] and, when revisited, led to mourning-filled contemplations of whom these early adults might have grown up to be, and the paths their lives would have taken, had there been no war. To some, these ongoing and distressing search processes were frequently concomitant with newly found purpose, intentional benefit-finding in the opportunities provided by life in resettlement, and perceived growth. This finding suggests that individuals with layered meaning systems, subject to cumulative PTEs that differentially violate specific cognitions [37,38], and with unresolved meaning searches can experience both growth and distress throughout their meaning-making trajectories [39].

Much as with the war, shattered beliefs and goals also required reappraisals at different points in the journey, thus confirming our two initial propositions that meaning-making is both a trajectory and a dynamic process that can be triggered by new violations or by a setting that allows survivors time to make meaning of their pasts. In the early months and years after resettlement, refugees are often focused on the daily challenges of adjusting to their new lives with little ability to address past losses and trauma and to engage in difficult reflections [40]. This form of avoidant coping, which can temporarily be adaptive despite associated poorer mental health outcomes [41,42], effectively delays cognitive processing until survivors reach a more favorable social context. In our sample, Portugal was generally reported as a welcoming and safe country. At the height of the 2015 European refugee crisis, communities across Portugal had eagerly readied themselves to host arriving refugees [43], and this welcoming context may have provided the conditions for arriving Syrians to reappraise the world and the self in a positive light. Participants’ ambivalent reappraisals of worldviews regarding safety, benevolence, and fairness, which were frequently prefaced by cautious statements related to geographical location, luck or other life circumstances, thus suggest that place and host community not only inform meaning-making processes but also provide sources for meaning itself [44].

Considering the centrality of religion to Syrian identity, group belonging, and place in society [35], we were surprised by the non-salience of religious themes in our findings. This may have been in part due to the study’s focus on meaning as socially and collectively construed, and to participants being recently arrived and not yet available to engage in more existential meaning-making. Of note, the role religion played in severing community ties during the war and the loss of trustworthy religious leadership may have effectively robbed participants of an important dimension of within-group coping [21,45]. Because religious beliefs generally offer relatively stable and accommodating cognitive structures [20,46], it is possible that, with time, individuals will further change their perceptions of the war and circumstances of displacement to fit pre-war religious structures. Yet the extent to which religion may have informed this sample’s cognitive reappraisals of both PTEs and global meaning thus far remains unknown. To help close this gap, future studies should investigate Syrians’ religion-informed trajectories of meaning-making as intra- and inter-personal processes and explore the impact of coping strategies and place of settlement on post-migration religious identity, rituals, and practices.

In our study, participants’ subjective negative reappraisals about their own community remained unresolved and a source of significant distress, which reinforced the uprootedness of the displacement experience and perceived lost sense of belonging [10]. Despite unsuccessful attempts by host organizations in Portugal to create spaces for dialogue and healing, it is possible that the ongoing war may have prevented students’ ability to begin repairing the shattered cognitions that informed the collective self. The absence of subnarratives related to social support in resettlement was also surprising given the significance of the community to Syrians’ identity and the role of social support is fostering adaptive meaning-making [47,48]. This void in the narratives may both justify individuals’ persistent negative reappraisals of beliefs about the community (i.e., meaning-making as output), as well as be conducive to maladaptive psychological adjustment (i.e., meaning-making as process).

Although it is possible that the ongoing war and recent arrival to Portugal may have contributed to incomplete meaning-making processes at the time of data collection, the coexistence of negative, positive, and ambivalent meanings-made points to meaning-making in the aftermath of refugee trauma as a dynamic process with no defined end, where collective identity and narrative are reshaped both over time and across generations [26,27]. Living free and in peace in resettlement provided our youthful, single, and student-majority sample ample opportunities for agency and growth. The goal of working towards a university degree may have been protective against some of the challenges of forced migration [49] and greatly informed students’ subjective sense of purpose, despite often struggling with overwhelming sense of responsibility and guilt over deriving any benefit from such a devastating event as the war. Increased compassion, changed priorities, and preoccupation with the common good, whether by using their newly acquired skills towards post-war reconstruction or giving back to both home and host communities, may provide pathways to restore a lost sense of collective self and promote posttraumatic growth [50].

Thus far, mental health research with Syrian refugees has mostly centered on negative psychological outcomes of exposure to collective trauma for the self and the community [51]. This qualitative study focused on eliciting community-informed meaning-making processes that conduce to positive psychological adjustment. The study makes important contributions to the literature that stress the decisive role that host communities can play in promoting adaptive adjustment when refugees are provided with safe opportunities for self-realization. Although the Syrian war disrupted community ties, reversed family roles, and led to the loss of home, country, humanity, and dignity [10,37], our findings suggest that the need some participants felt to seek deeper meaning in their experiences through purposive meaning-making processes may have led them to perceiving growth in their restored selves. Manifestations of posttraumatic growth included increased empathy and courage, new possibilities, sense of pride in their journeys, and greater appreciation for life [16] that align with recent studies with Syrian refugees [52]. In line with the posttraumatic growth literature, these subjective positive changes require a significant level of shattering of previous cognitions, which the war provided, and the subsequent integration of the past into a coherent narrative [53]. This process was incisively articulated by one participant as the need he felt to respect his own experience, which “built” him.

Our work makes important contributions to the meaning-making literature by introducing a trajectory analysis of meaning-making from pre-trauma global meaning through post-settlement psychological adjustment. Theoretical propositions suggest that meaning-making occurs within the context of the culture and the community [48], which is especially important for individuals belonging to collectivist societies and survivors of community-impacting trauma. By focusing on community and collective processing and analyzing meaning as a journey, our findings suggest that: meaning-making is dynamic and occurs at different points throughout displacement according to individual and community circumstances and lived experiences; the integrated meaning-making model is robust and sufficiently comprehensive and flexible to capture the experiences of non-Western survivors of collective, cumulative trauma; and survivors of refugee trauma can make both adaptive and maladaptive reappraisals of cognitive-specific stressors, which subsequently contribute to coexisting positive and negative psychological adjustment.

This study is not without limitations. Participants were interviewed eight years after the onset of the war, therefore their retrospective recollections of pre-war and wartime beliefs and goals are likely influenced by multiple biases, including individually experienced traumas, displacement journeys, and life circumstances. Further, although in some ways having a sample comprised primarily of student refugees was advantageous in terms of participants’ abilities to give in-depth accounts of their meaning-making processes, this group’s experiences may not be representative of those of other war-exposed Syrians living in Portugal, despite the sample’s geographic diversity and our attempts to include Syrian nationals regardless of legal status. The interviews were not conducted in participants’ native Arabic, which may have posed difficulties for participants to fully and accurately express the nuances of their meaning-making efforts. Lastly, this study only focused on the impact of the war as baseline for meaning violation and on meaning trajectories related to the collective, and hence does not represent the totality of individuals’ meaning-making experiences, including the wealth of individually-experienced traumas, intrapersonal reappraisals, and coping strategies, namely those related to religion, faith, and spiritual dimensions of healing, that may facilitate or hinder adjustment. Future research should investigate intrapersonal meaning-making trajectories in the aftermath of the collective trauma, including reappraisals in transit countries, to comprehensively capture refugees’ journeys in meaning.

## 5. Conclusions

The Syrian war has profoundly and critically severed community ties and Syrians’ collective identity. Adaptive meaning-making of those shattered cognitive structures will require ongoing and iterative reappraisals, possibly over generations, as displaced Syrians attempt to make sense of the war, find benefit in their experiences, and rebuild narratives that can restore their lost sense of self. Host communities are key to promoting adaptive psychological adjustment, by providing: welcoming and safe places that help repair refugees’ lost sense of humanity and rebuild community trust; designing host programs that incorporate opportunities for self-realization and purpose (e.g., access to higher education or to meaningful jobs); and providing healing opportunities for within- and inter-community exchanges that help facilitate meaning-making and psychological growth in survivors of refugee trauma.

## Data Availability

The datasets generated and analyzed during this study include a large qualitative component and are not publicly available.
